# Foreign Travel

**Published:** 1873-01

**Authors:** 


### FOREIGN TRAVEL.

In winter, as well as in summer, there are long lists of passengers in the various steamship lines for the old world. We received, the other day, a cordial letter of thanks from a stranger, for the valuable practical suggestions to travelers going abroad, contained in some previous issue of the Journal. "Where to put up at," so as to secure comfort at moderate prices, is a question which multitudes would like to have satisfactorily answered. Almost every intelligent traveler visits quaint old Heidelberg, one of the most interesting places in Europe. At Herr Long's Hotel, the traveler will always meet with the courteous and prompt and accommodating attention of the proprietor, who speaks English and several other languages with great fluency; he has been quite a traveler himself, and can give a large amount of useful information. The hotel, which he owns, is convenient to the railway station.

Madam Föstor, on the Anlage, the most beautiful promenade of the place, receives ladies and children; any one stopping with her is sure to leave with regret, and will have pleasant memories ever thereafter. Should a physician be required, the most skillful and patiently attentive are Dr. Mittermeyer and Dr. Von Tuch.

At Naples, a pleasant and agreeable home will be found under the roof of an English lady, Mrs. Turner Guidotti, 64 Vico Carminello a Chiaja, with the cordial co-operation of her courteous and gentlemanly husband. Dr. Sammut is the most able and eminent medical practitioner, and is a great favorite among both English and Americans.

At Dresden, Mrs. Hughes, at 34 Prager Strasse, who speaks both German and English fluently, keeps the very best American-like boarding house; her American cookery, American abundance and tidiness in every department of her household, with the elegant surroundings, combine to draw to her a great many English and Americans.

Of course Paris is the Mecca of every traveler; to go abroad and not to see Paris, is not to have traveled at all. The energy, and forethought, and enterprise of Louis Napoleon have made it akin to an earthly paradise. But to enjoy Paris, you must have a comfortable home, and at a reasonable expense; and to have a landlady who is a lady in heart as well as in manners, who will look out for your comfort for the sake of making you comfortable, is a very great boon; such an one will be found in Mlle. Bocquet, 72 Boulevard Neuilly. Her charges are very moderate; she has a houseful of young ladies from all parts of "the States" and England, who are desperately bent on learning how to speak French; she is not a teacher herself, but knowing the importance of compelling one to speak French as a means of learning it, she rigidly adheres to the rule of knowing only French under her roof. Fortunate are they who can find a vacant place at her table, and around her fireside

